# Palpable Purpura with an Unexpected Outcome

**DOI:** 10.1155/2013/678427

**Published:** 2013-07-15

**Authors:** Anne Friesgaard Christensen, Ole Clemmensen, Peter Junker

**Affiliations:** ^1^Department of Rheumatology, Odense University Hospital, Sdr. Boulevard 29, 5000 Odense, Denmark; ^2^Department of Clinical Pathology, Odense University Hospital, Sdr. Boulevard 29, 5000 Odense, Denmark

## Abstract

Scurvy is a potentially lethal condition caused by inadequate vitamin C intake. Hypovitaminosis of vitamin C causes vessel fragility and follicular hyperkeratosis that can lead to palpable purpuric skin lesions. In this case report, we aim to remind readers that scurvy still occurs in Western countries and that the clinical presentation may resemble vasculitis.

## 1. Introduction

Scurvy is a potentially lethal condition caused by inadequate vitamin C intake. It is rarely diagnosed in Western countries, although vitamin C deficiency has been estimated to occur in up to 17% of hospitalized patients [1]. Similarly, data from the National Health and Nutrition Examination Survey suggested a prevalence of vitamin C deficiency in the US at 10–14% in adults [2]. Since purpuric skin and mucosal lesions are an early hallmark, scurvy may be misconceived as a systemic vasculitis. Recognition of some distinctive features may facilitate early diagnosis and treatment. 

## 2. Case

**Figure 1:**
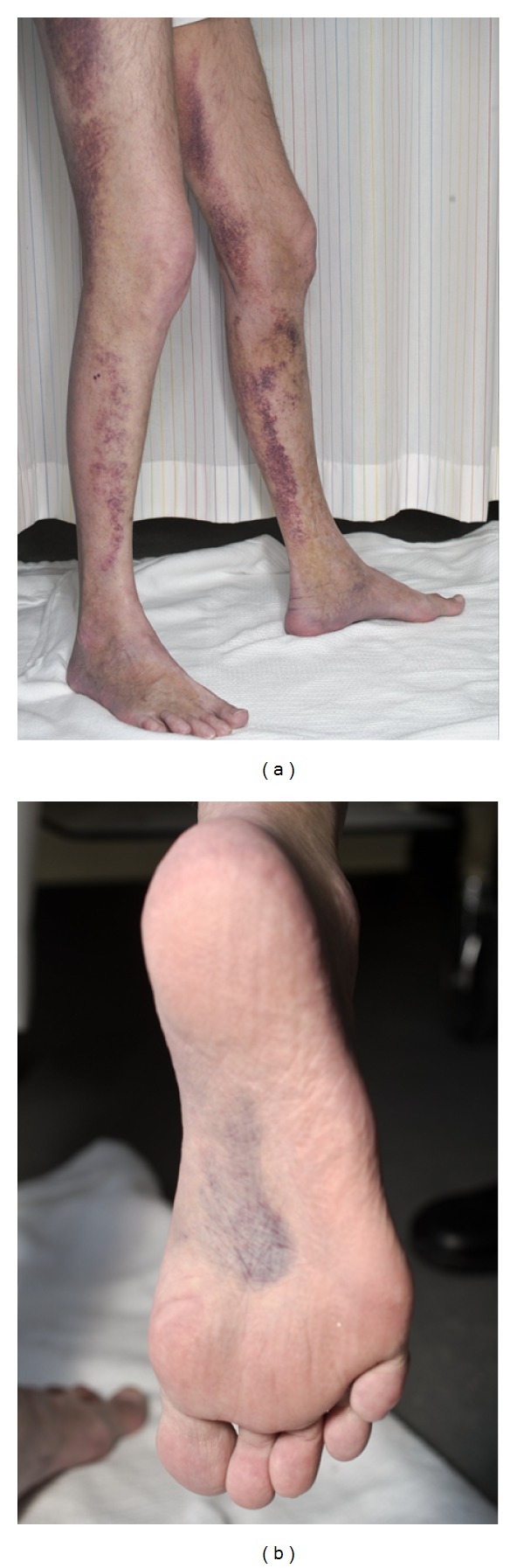
Palpable, purpuric rash and confluent ecchymoses on both legs and in the soles.

A 49-year-old Caucasian male was admitted for vasculitis. For more than 10 years, he was addicted to heroin, recently substituted by Methadone. He denied intravenous drug abuse. During the past 6–12 months, he had experienced loss of physical performance due to aching legs, decreasing muscle strength, weight loss, malaise, and fatigue. He had virtually no outdoor activities, and his preferred diet was white bread with chocolate butter. One week before admission, he noticed nonulcerating, painful purpuric rash on both legs (Figure 1). There had been no fevers, ear-nose-throat manifestations, lung symptoms, abdominal pain, Raynaud's phenomenon, mucosal bleedings, or ulcerations. The patient did not take any medications apart from methadone, and had no exposures to insects or any travels abroad. On physical examination, he appeared in a poor condition, slightly anemic, blood pressure 125/92 mmHg, temperature 37,4 Celius, peripheral pulse 100 beats/min, respiratory rate 18/min, weight 65 kg, and height 179 cm. He presented with reduced locomotor function due to painful legs, calves in particular. A palpable, purpuric rash and confluent ecchymoses were noted on both legs, most pronounced at the medial aspects of thighs and lower legs (Figure 1). There were scattered purpuric elements on the buttocks, but no proximal lesions. Walking and even mild physical exercise resulted in severe worsening of pain and progression of purpura into ecchymoses (Figure 1). During the following days, suggilations appeared in the soles (Figure 1).

**Figure 2:**
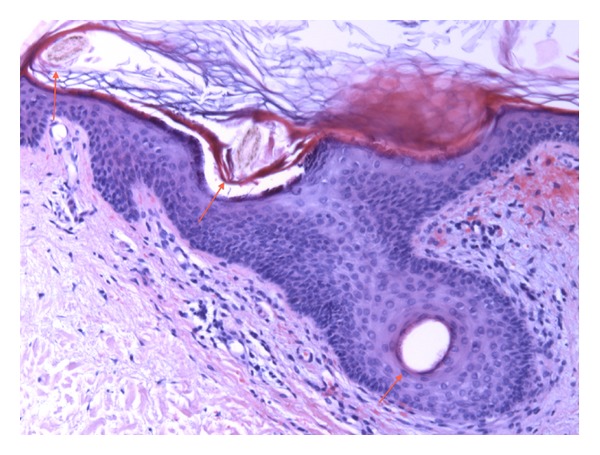
A punch biopsy from the right lower leg shows a sparse, perifollicular, lymphocytic infiltrate and extravasation of erythrocytes. There is hyperkeratosis of the slightly dilated follicular infundibulum. The two transverse sections of the intracorneal parts of a hair shaft (arrows) show the spiraling course of the hairs (cork screw appearance) characteristic of scurvy.

 B-hemoglobin was 5,2 mmol/L, and mean corpuscular volume (MCV) was 111 *μ*³ (80–100). The white blood cell and platelet counts were normal. C-reactive protein (CRP) was 87 (<10) mg/L. Creatinine and liver function tests were normal, except for slightly increased alkaline phosphatases, 138 U/L (35–105). Coagulation screening was unremarkable, and P-albumin was 35 g/L. Creatine kinase and urine analyses were normal. S-IgA was 3,76 g/L (0,70–3,65), and there were normal levels of IgG and IgM. There was no M-component. He tested negative for hepatitis B and C, human immunodeficiency virus (HIV), cryoglobulin, IgM-rheumatoid factor, anti-nuclear antibodies (ANA), anti-neutrophil cytoplasmic antibodies (ANCA, proteinase-3, and myeloperoxidase), and phospholipid antibodies. Complement levels were normal. There were no opacities on Chest X-ray. Blood cultures were negative. A skin biopsy showed erythrocyte extravasation and follicular hyperkeratosis, but no vasculitis, complement or immunoglobulin deposits (Figure 2).

Additional analyses revealed low 25-hydroxy-vitamin D, <6 mmol/L (50–160), ionized calcium, 116 mmol/L (1,19–1,29), S-folate, <3,4 nmol/L (5,0–30,0), elevated parathyroid hormone, 13,1 pmol/L (1,10–6,50), and normal S-cobalamin. Ascorbate in serum was barely detectable, <3,0 *μ*/L (26,1–84,6).

Replenishment with vitamins C and D, folic acid, and calcium and adjustment of his dietary habits were followed by rapid and sustained recovery. One month later, serum levels of calcium and vitamins were normal with S-ascorbate at 30,5 *μ*/L.

## 3. Discussion

The presenting manifestations of scurvy in this case included several features shared by the systemic vasculitides including palpable purpuric rash, myoskeletal pain, weight loss, and fatigue. Despite drug addiction, hepatitis C-associated cryoglobulinemia was excluded. There was no evidence of septic or cholesterol microembolism, antiphospholipid syndrome, or other coagulopathies. Symmetric purpura affecting the lower extremities and elevated IgA in serum indicated adult Henoch-Schönlein purpura, but this was ruled out by a skin biopsy compatible with hypovitaminosis C (Figure 2). The impact of dermal and muscle microvascular fragility was further evidenced by the emergence of plantar suggilations following physical exercise.

While inflammation is the cause of vessel damage in vasculitis, increased small vessel fragility due to collagen compromise is the hallmark of the scurvy. Thus, ascorbate is an essential cofactor for prolyl-4-hydroxylase which is a prerequisite for hydroxylation of procollagen *α*-chains and hence formation of triple helical collagen and fibrillogenesis [3, 4].

Contrary to purpura due to scurvy, vasculitic purpura is usually palpable. Of note, however, purpuric lesions in scurvy may also be palpable due to follicular hyperkeratosis and perifollicular hemorrhage as observed in the present case. Anemia is a frequent finding in scurvy, usually microcytic due to inadequate iron absorption [5]. However, anemia may be of mixed origin, in this case due to folic acid deficiency reflecting a more general shortage of micronutrients including vitamin D and calcium. 

This case illustrates that scurvy should be considered in patients presenting with painful, purpuric skin lesions and aching muscles with worsening upon physical activity. Further clues include poor dietary habits, multiple micronutrient deficiencies, and brittle dermal microvessels on biopsy without vessel wall inflammation. The diagnosis is established definitely from low ascorbate serum level and complete recovery from substitution therapy.
